# Prediction of extubation failure in newborns, infants and children: brief report of a prospective (blinded) cohort study at a tertiary care paediatric centre in India

**DOI:** 10.1186/s40064-015-1607-1

**Published:** 2015-12-30

**Authors:** Bedangshu Saikia, Nirmal Kumar, Vishnubhatla Sreenivas

**Affiliations:** Department of Paediatrics and Neonatology, St Stephens Hospital, Tis Hazari, New Delhi, 110054 India; Department of Biostatistics, All India Institute of Medical Sciences, New Delhi, 110029 India

**Keywords:** Extubation failure, Respiratory effort, Poor cough reflex, Thick secretions, Spontaneous breathing trial, Rapid shallow breathing index

## Abstract

**Background:**

Extubation failure (EF), defined as need for re-intubation within 24–72 h, is multifactorial. Factors predicting EF in adults generally are not useful in children.

**Objective:**

To determine the factors associated with EF and to facilitate prediction of EF in mechanically ventilated infants and children less than 12 years of age.

**Material and Methods:**

*Design* Prospective cohort study. *Setting* PICU and NICU of a multispecialty tertiary care institute. *Patients* All consecutive newborns, infants and children, who remained on the ventilator for more than 12 h, were included. Patients with upper airway obstruction, neuromuscular disorders, complex anatomic malformations, accidental extubation, tracheostomy or death before extubation were excluded. *Methods* The pre-extubation clinical, laboratory and ventilatory parameters were collected for 92 cases over a one and half year period. The EF rate was calculated for each variable using STATA 9. All the treating physicians were blinded to the data collection procedure.

**Measurements and Results:**

Demographics were comparable between the extubation success and EF groups. Respiratory failure was the main cause requiring ventilation (46.74 %, 95 % CI 0.37–0.57) as well as EF (30.23 %, 95 % CI 0.08–0.23). 76.92 % (95 % CI 0.58–0.89) of patients that failed extubation had alterations in respiratory effort, 38.46 % (95 % CI 0.22–0.57) each had either poor or increased respiratory effort. Poor cough reflex (p = 0.001), thick endotracheal secretions (p = 0.02), failed spontaneous breathing trial (SBT) (p = 0.001) and higher rapid shallow breathing index (RSBI) (p = 0.001) were found to be associated with EF.

**Conclusions:**

Paediatric EF is multifactorial. Increased or poor respiratory effort and failed SBT are potential factors in deciding re-intubation. Increased RSBI, poor cough reflex and thick.

## Background

After resolution of illness, mechanically ventilated patients are disconnected from the ventilator; extubation is the final step in this process. Extubation failure (EF) is defined as an inability to sustain spontaneous breathing and need for re-intubation within 24–72 h after extubation (Rothar and Epstein 2003). Prediction of EF is essential, as both delayed and failed extubation have detrimental consequences (Rothar and Epstein 2003). The incidence of EF varies between 2 to 47 % (Rothar and Epstein 2003; Kulkarni and Aggarwal 2008). It can be as high as 22 to 28 % in premature neonates (Khan et al. 1996).

A variety of patho-physiologic causes lead to EF. The prediction of EF is difficult (Rothar and Epstein 2003; Kulkarni and Aggarwal 2008). Newth et al. reported limited guidance on paediatric weaning and extubation from their literature review (Newth et al. 2009). Indices developed to predict weaning and extubation success (ES) are no better than clinical judgment (Newth et al. 2009). Similarly, Leclerc and Schindler observed that adult weaning predictors proposed by the Task Force of the American College of Chest Physicians have very poor predictive power in children (Yang and Tobin 1991; Leclerc et al. 2005; Schindler. 2005).

We undertook this study in newborns, infants and children to explore factors that may predict EF.

## Methods

This prospective cohort study was conducted at PICU and NICU of a multispecialty tertiary care institute in India over a period of one and half years (December 2008 to May 2010). All consecutive infants and children less than 12 years of age, admitted and ventilated for more than 12 h were included in the study. Patients with upper airway obstruction, accidental extubation, tracheostomy, or death before extubation were excluded. The sample size was calculated using 95 % confidence interval, 10 % margin of error and 30 % estimated incidence of EF in the study area. We did a retrospective analysis of PICU register books to find out the incidence of EF in our PICU. We looked at the previous 2 years data and approximated the incidence of EF in our PICU to be around 30 %. We used the formula, *n* *=* *t*^*2*^*x p (1−p) x 1/m*^*2*,^ where ‘*n*’ is required sample size, ‘*t*’ is confidence level at 95 %, ‘*p*’ is estimated incidence of EF in the project area and ‘*m*’ is margin of error at 10 % (Calculating the sample size—IFAD). This gave us the minimum sample required for the study as 81 and we collected data for 92 patients. The research team was not involved directly in the clinical care. All decisions related to patient’s care were taken by the treating physician and they were blinded to data collection and analysis procedure.

The parameters that were collected were divided into: (1) *Demographic data*: Age, sex, weight, diagnosis on admission, indication for ventilation and duration of intubation, (2) *Clinical parameters*: Haemoglobin concentration, heart rate, spontaneous respiratory rate, blood pressure, peripheral oxygen saturation (SpO_2_), work of breathing, presence of cough reflex, amount and consistency of secretion, use of ionotropes, use of sedation and use of dexamethasone, (3) *Blood gas parameters (venous gases)*: pH, partial pressure of carbon dioxide (PCO_2_), bicarbonate (HCO_3_^−^), base excess (BE), lactate and (4) *Ventilatory parameters*: Ventilator mode, ventilator rate, peak inspiratory pressure (PIP), positive end-expiratory pressure (PEEP), fraction of inspired oxygen (FiO_2_), inspiratory time (Ti), expiratory time (Te), spontaneous breathing trial (SBT), rapid shallow breathing index (RSBI) and use of bubble CPAP following extubation. These parameters were measured pre-extubation, post-extubation (whenever necessary), and at the time of any re-intubation.

### Derived parameters

Indices that incorporated more than one measurement of respiratory function i.e., mean airway pressure {MAP = [(PIP − PEEP) (Ti)/Ti + Te] + PEEP} and ventilator index {VI = [Ventilatory rate × (PIP − PEEP)*PCO_2_]/1000} were calculated.

### Data analysis

EF rate was calculated for each variable using the statistical software STATA 9, version 17. Data was presented in frequency percentage with confidence intervals and mean (SD) and median (minimum–maximum). In the continuous parameters, average (mean/median) between the two groups was compared by using t test and Wilcoxan rank sum test. In the categorical variable, two groups were compared by using Chi-square and Fisher’s exact test. p value <0.05 was taken as significant.

### Ethics, consent and permissions

Institute’s ethics committee approval was obtained and patients were recruited after written informed parental consent.

### Consent to publish

Parental permissions were obtained for presentation of study results in conferences and also publication in journals.

## Results

Two hundred and eleven infants and children were ventilated during the study period. 119 patients could not be included into the study (38 did not meet inclusion criteria, 46 died while on ventilator, 19 left hospital against medical advice and 16 patients referred out). 92 patients were included into the study; 66 patients successfully extubated (success rate 71.7 %, 95 % CI 0.62–0.79) whereas 26 patients failed extubation (failure rate 28.3 %, 95 % CI 0.21–0.38). The demographic data of the two groups are shown in Table 1.

**Table 1:** Demographics of the two groups

Parameters		Group A (extubation success; n = 66)	Group B (extubation failure; n = 26)	Probability (p value)
Age (category wise)	Birth–1 month	40	15	0.617
	1–12 months	12	7	
	1–12 years	14	4	
Weight in kg (mean ± SD)	Birth–1 month	2.39 ± 0.92	2.02 ± 1.02	0.942
	1–12 months	4.33 ± 1.51	4.16 ± 1.53	
	1–12 years	17.93 ± 7.23	13.95 ± 6.35	
Gestational age of newborns *(NB: All babies <35 received ante natal corticosteroid therapy in the form of maternal betamethasone)*	Term (37 weeks and above)	19	7	0.07
	Pre-term (35 to <37 weeks)	5	0	
	Pre-term (32 to <35 weeks)	8	1	
	Pre-term (<32 weeks)	5	6	
Neonate (birth–1 month) with birth weight (kgs) *(NB: All preterm babies <37* *weeks weighed <2.5kgs)*	≥2.5 kgs	17	6	0.867
	<2.5 kgs	23	9	
System involved	Respiratory and cardio–respiratory	31	15	0.711
	Multisystem	14	4	
	Others	21	7	

Table 2 shows diagnosis of patients requiring mechanical ventilation. Most common indication of ventilation was pneumonia and sepsis accounting for 14 (15.2 %) cases each. In the neonatal category, most of babies were ventilated for hyaline membrane disease, birth asphyxia, meconium aspiration syndrome and congenital pneumonia, accounting for 11 (11.9 %), 8 (8.7 %), 7 (7.6 %) and 7 (7.6 %) cases respectively. Post-operative state accounted for 9 (9.8 %) cases, whereas 8 (8.7 %) cases were ventilated as a part of the management protocol in cases of refractory status epilepticus. Others being acute respiratory distress syndrome [3 (3.3 %) cases], acute bilirubin encephalopathy [1 (1.1 %) case], bronchiolitis [2 (2.2 %) cases], CNS bleed (vitamin K deficiency bleed) with seizures [1 (1.1 %) case], congenital diaphragmatic hernia (right) [1 (1.1 %) case], congenital heart disease with cardiac failure [1 (1.1 %) case], congenital pneumothorax [1 (1.1 %) case], refractory apnea of prematurity [1 (1.1 %) case], refractory status asthmaticus [2 (2.2 %) cases] and viral meningoencephalitis [1 (1.1 %) case].

**Table 2:** Diagnosis of study cases along with total number in each category (percentage of total) for which ventilation was needed

Diagnosis	No of cases (%)	Diagnosis	No of cases (%)
Acute bilirubin encephalopathy	1 (1.1)	Meconium aspiration syndrome with PPHN	7 (7.6)
Acute respiratory distress syndrome	3 (3.3)	Pneumonia	14 (15.2)
Bronchiolitis	2 (2.2)	Post-operative state	9 (9.8)
CNS bleed (VKDB) with seizures	1 (1.1)	Refractory apnea of prematurity	1 (1.1)
Congenital diaphragmatic hernia (Right)	1 (1.1)	Refractory status asthmaticus	2 (2.2)
Congenital heart disease with cardiac failure	1 (1.1)	Refractory status epilepticus	8 (8.7)
Congenital pneumonia	7 (7.6)	Severe birth asphyxia	8 (8.7)
Congenital pneumothorax	1 (1.1)	Severe Sepsis	14 (15.2)
Hyaline membrane disease	11 (11.9)	Viral meningoencephalitis	1 (1.1)

Of the total 92 ventilated patients, 43 had pulmonary involvement (46.74 %, 95 % CI 0.27–0.57), 13 failed extubation (30.23 %, 95 % CI 0.18–0.45). 19 patients had central nervous system involvement (20.65 %, 95 % CI 0.14–0.31), four (21.05 %, 95 % CI 0.08–0.43) had EF. Multisystemic involvement (more than one system involvement) was seen in 18 patients (19.56 %, 95 % CI 0.13–0.29); four had EF (22.22 %, 95 % CI 0.1–0.45). Nine (9.78 %, 95 % CI 0.05–0.18) were ventilated post-operatively; three (33.33 %, 95 % CI 0.12–0.65) failed extubation. Three (3.26 %, 95 % CI 0.01–0.1) cases were ventilated for cardio–respiratory system involvement; two (66.67 %, 95 % CI 0.21–0.94) failed extubation. The patients were divided into various categories depending upon the involvement of system; multisystemic refers to more than two system involvement.

Out of the 26 cases with EF, 10 each 38.46 % (95 % CI 0.22–0.57) had either poor or increased respiratory effort; therefore alteration in the respiratory effort accounted for 76.38 % (95 % CI 0.58–0.89) of EF (two each had recurrence of seizures and post seizure respiratory arrest respectively, one developed pneumothorax and another one had recurrence of apnoea of prematurity).

We observed that poor cough reflex contributes to EF. In the EF group, one patient had good cough reflex and the rest 25 had poor cough reflex, whereas in the ES group, 33 had good cough reflex and the other 33 had poor cough reflex, p = 0.001. We also observed that thick secretions contributes to EF as well, 15 patients in the EF group and 21 patients in the ES group had thick secretions (p = 0.02), whereas the amount of secretion was not significant, p = 0.12. Another observation was—patients who passed 30 min SBT were successfully extubated (n = 48), whereas all patients who failed SBT and extubated subsequently had EF (n = 7); p = 0.001. Our study also showed that a higher RSBI was associated with EF, p = 0.001, 91 in ES vs 169 in EF Details in Table 3.

**Table 3:** Study results

Parameters	Group A (extubation success; n = 66)	Group B (extubation failure; n = 26)	Probability (p value)
Cough reflex			
Good	33	1	0.001
Poor	33	25	
Consistency of secretion			
Thick	21	15	0.022
Thin	45	11	
Amount of secretion			
Plenty	7	6	0.122
Minimal	59	20	
Sedation			
Yes	45	13	0.104
No	21	13	
Ionotropes			
Yes	49	18	0.627
No	17	8	
Spontaneous breathing trial (SBT)			
Passed	48	9	0.001
Fail	0	7	
Spontaneous respiratory rate (per minute)	31 (8–90)^a^	37 (8–76)^a^	0.298
Base excess	−1.7 (−18.8–6.6)^a^	−3.3 (−10.3–1.6)^a^	0.017
Serum lactate	1.7 (0.6–6.6)^a^	1.7 (0.7 5.1)^a^	0.883
Expiratory time	2.6 (0.91–9.55)^a^	2.6 (0.95–11.5)^a^	0.872
Duration of ventilation (in hours)	66 (13–314.5)^a^	52.5 (13–247.5)^a^	0.422
Rapid shallow breathing index (RSBI)	91 (58–173)^a^	169 (67–564)^a^	0.001
Hemoglobin (gm/dL)	12.96 ± 2.82^b^	12.68 ± 3.13^b^	0.679
SpO_2_	96.68 ± 2.32^b^	96.54 ± 3.06^b^	0.809
pH	7.38 ± 0.07^b^	7.36 ± 0.06^b^	0.339
PaCO_2_	37.64 ± 6.84^b^	37.65 ± 6.82^b^	0.994
Bicarbonate (HCO_3_)	21.64 ± 3.96^b^	20.89 ± 3.16^b^	0.397
Inspiratory time	0.52 ± 0.16^b^	0.49 ± 0.12^b^	0.444
Positive inspiratory pressure (PIP)	14.71 ± 2.72^b^	15.08 ± 1.87^b^	0.533
Positive end expiratory pressure (PEEP)	5.68 ± 1.78^b^	5.78 ± 0.81^b^	0.729
Fraction of inspired oxygen (FiO_2_)	0.31 ± 0.09^b^	0.33 ± 0.12^b^	0.391
Mean airway pressure (MAP)	7.13 ± 1.32^b^	7.29 ± 1.14^b^	0.769
Ventilator index (VI)	6.94 ± 4.49^b^	6.94 ± 4.49^b^	0.996
Continuous positive airway pressure (CPAP)	6.11 ± 0.78^b^	6.11 ± 0.32^b^	1.0

[Cough reflex (p = 0.001) and thick secretions (p = 0.022) has shown strong relationship extubation failure. We also found that all patients who passed SBT were successfully extubated, whereas all patients who failed SBT ultimately failed extubation (p = 0.001). Similarly higher RSBI was associated with extubation failure (p = 0.001)]

N.B.: ^a^ median values (minimum–maximum)

^b^ mean value ± SD

Please see Appendix 1 for definitions used in this study.

## Discussion

We found that paediatric EF could be multifactorial. We considered a host of different factors which could possibly cause EF as observed in previous studies. We found that respiratory system involvement, failure to pass SBT and altered respiratory effort following extubation are major concerns towards EF as well as poor cough reflex and thick secretions. The major challenge we encountered was paucity of data from this part of the world on paediatric EF. Paediatric and neonatal EF is a very well addressed issue in the resourced countries unlike in the resource poor countries and therefore we think it would be unwise to compare our findings with the studies done previously in resourced countries. At the same time we would like to say, this is the first kind of study on EF from the entire Indian subcontinent including all age groups and variety of factors.

Several patient characteristics have been implicated in paediatric ES and EF (Rothar and Epstein 2003; Kulkarni and Aggarwal 2008). As compared to most neonatal and paediatric ICU’s in developed countries, we found a high EF rate, 28.3 % (95 % CI 0.21–0.38). Previous studies show incidences separately for neonatal and paediatric extubation failures. Our study population was a mixed one, but surprisingly we could not find any intergroup difference (0–1 m vs 1–12 m vs 1–12 year). Moreover, we could not find any data on neonatal or paediatric EF rates from Indian subcontinent to compare with.

In the current study, we noticed that 50 % of the children who failed extubation had a pre-morbid respiratory system involvement. EF amongst post-operative cases were possibly because of premature weaning and extubation by the treating team. Irrespective of the system involvement more than 3/4th (76.92 %) of the patients who failed extubation had a change in the respiratory effort (poor or increased). It has been shown in previously that paediatric EF is in part disease specific and pre-existing respiratory conditions predispose to re-intubation (Kurachek et al. 2003).

Prematurity, low birth weight, younger age, prolonged duration of ventilation, CPAP after extubation, use of inotropes, sedation and analgesia are known to contribute to EF, but our study failed to show any difference (Fontela et al. 2005; Hiremath et al. 2009; Epstein 2002a, 2002b; Dimitriou et al. 2002; Stawicki. 2007). Post-extubation nasopharyngeal bubble CPAP (Kaur et al. 2008) was used mostly for cases with respiratory system involvement. Likewise, poor oxygenation is an established risk factor for re-intubation. We could not look into A-a gradient, PaO_2_/FiO_2_ ratio and oxygenation index although it was planned. In our ICU setting most clinicians prefer venous blood gases (VBG) for determining acid–base status of the body as pH, PCO_2_, HCO_3_ and BE are comparable in both venous and arterial blood gases (Ahmet et al. 2006; Chu et al. 2003) and also relatively easy to perform in resource poor setting. We, in this study could not find any difference in terms of blood gas and ventilatory parameters in the two groups.

In the current study, we found that failure of SBT has a strong correlation with EF (p value 0.001). As a measure of extubation readiness, our ITUs have a policy of performing a 30 min SBT in in our NICU and PICU patients; the same protocol as that for a 2 h SBT, as suggested by Randolph et al. was followed but with adaptable modification (Curley et al. 2006; Venkataraman 2006; Thiagarajan et al. 1999). When found to have no clinical need for increased ventilatory need in the previous 12 h, spontaneously breathing, good effort of breathing, good tidal volume (VT) and SpO_2_ >95 %, child was started on SBT after stopping feeds and titrating sedation to minimum or stopped at least 4 h beforehand. Ventilator settings changed to flow triggered CPAP–PSV mode with FiO_2_ 0.5, positive end expiratory pressure (PEEP) 5 cm H_2_O and pressure support (PS) as per the size of endotracheal tube (ETT); 10 cm H_2_O if ETT 3–3.5 mm; 8 cm H_2_O if ETT 4–4.5 mm; and 6 cm H_2_O if ETT ≥5 mm). SpO_2_, exhaled VT and respiratory rate (RR) monitored and if found to be in the target range at the end of 30 min, child was declared to pass SBT and prepared for extubation, otherwise patients were put back on the same pre-test ventilator settings. [Targets: SpO_2_ ≥95 %, exhaled VT ≥5 ml/kg and RR-<6 month, 20–60/min; 6 month–2years, 15–45/min; 2–5years, 15–40 and >5years, 10–35/min].

In our study, SBT was performed in 64 study patients. 48 patients passed SBT and all of them were successfully extubated. In EF group, nine patients passed SBT and extubated but eventually they failed extubation; whereas seven patients were extubated despite they failed SBT and all of them failed extubation. Farius and Kamlin also observed a similar association in their study (Paret et al. 1998; Kamlin et al. 2006). We also observed RSBI as a potentially useful index that can predict EF—the higher the value, the higher the chances of EF (ES 91 vs EF 169, p = 0.005). Previously, role of RSBI was described as controversial. Some researchers concluded it as a good indicator (Paret et al. 1998; Baumeister et al. 1997) whereas some labelled RSBI as a poor predictor (Farias et al. 2002; Venkataraman et al. 2000; Leclerc et al. 2002). We also found that poor cough reflex and thick secretion are potential risk factors for EF (p value 0.001 and 0.022 respectively); we could not find any relationship with secretion volume and EF (p value 0.122), though we could not exactly quantify secretion volume due to operational problems. These were, although been described as risk factors for EF in previous studies (Epstein 2002a, 2002b).

In the current study, we could not observe any difference in the use of pre extubation use of dexamethasone in preventing EF (p = 0.632). Previously, adult studies have proven the benefit of pre extubation steroid use in preventing EF, but paediatric studies have shown mixed results (McCaffrey et al. 2009; Sinha et al. 2010; Matthew. 2008; Khemani et al. 2008).

## Limitation of the study

The consistency and amount of endotracheal secretions were subjective. Arterial blood gases would be a better parameter to use than venous blood gases (VBG). VBG was chosen as the pragmatic alternative.

The study of more specific ventilation variables like CROP (compliance, rate, oxygenation and pressure) (Paret et al. 1998; Farias et al. 2002; Venkataraman et al. 2000). Simplified weaning index (SWI), compliance of respiratory system (CRS), rapid shallow breathing occlusion pressure (ROP) (Kulkarni and Aggarwal 2008; Dimitriou et al. 2002; Paret et al. 1998). Maximal inspiratory pressure during an occlusion test (Pi_max_), modified tension timed index (TTI) (Matthew. 2008; Noizet et al. 2005; Jabour et al. 1991) and dead space to tidal volume ratio (VD/VT) (Harikumar et al. 2009) would have strengthened the study. As the measurement of these variables requires expertise and special instruments we were unable to perform the same.

## Conclusion

Paediatric EF may be multifactorial and in part disease specific. The measurement of respiratory effort and SBT could be vital parameters in deciding re-intubation. In addition, increased RSBI, poor cough reflex and thick secretions may augment prediction of EF. This study also forms the basis for future studies on this topic. We believe this study would attract neonatologist, paediatricians and intensivists especially from resource limited countries for further discussions and research on EF.

## Appendix

### Definitions of parameters that are being used in the study

#### Measurement of cough reflex

Patient’s cough reflex was assessed and graded as good or poor. As per the unit policy, a good cough reflex is one where patient produces vigorous cough with none to very minimal stimulation (e.g., during nebulisations or during suctioning of oro-pharyngeal secretions); otherwise the reflex is a poor one.

#### Measurement of amount of tracheal secretion

A note of amount of tracheal secretion in the previous 24 h before extubation was made and quantified as minimal or plenty depending upon requirement of tracheal suctioning by open suctioning system. Our paediatric and neonatal intensive care unit has a policy of tracheal suctioning once in every 6 h. If frequent suctioning of trachea (e.g., many times in an hour or hourly) was demanded because of secretions causing respiratory compromise in terms of oxygenation and ventilation, then the tracheal secretions were said to be ‘plenty’; otherwise tracheal secretions were labelled as minimal.

#### Measurement of consistency of tracheal secretion

The unit has a policy of labelling the tracheal secretions as thick or thin depending upon its stickiness to the suction catheter. Secretions were said to be thick when these blocks the suction catheter and were difficult to remove from the catheter lumen by simple measures like simply suctioning distilled water.

#### Definition and measurement of respiratory effort

We used Paediatric Advanced Life Support (PALS), American Heart Association, 2005 guidelines to assess respiratory effort of patients by observing the spontaneous respiratory rate, breathing pattern (deep or shallow breathing), use of accessory muscles of respiration, peripheral circulation (pulse rate and volume and/or change in colour) and SpO_2_. We defined poor respiratory effort as having bradypnea, bradycardia, pallor or cyanosis, desaturation, shallow breathing pattern and poor use of accessory muscles of respiration. Whereas increased respiratory effort was described as tachypnea, bradycardia or tachycardia, cyanosis, desaturation, deep or shallow breathing and excessive use of accessory muscles of respiration. Age and sex specific charts for respiratory rate and heart rate (as given in PALS guideline) were used to determine tachypnea/bradypnea or tachycardia/bradycardia.

#### Nasopharyngeal CPAP

It was delivered using a modified bubble CPAP machine developed at our unit (Kaur et al. 2008).

#### Extubation failure

In our study, we defined EF as need for re-intubation within 72 h of extubation.
